# Turnover‐Dominated β‐Diversity and Its Temperature and Trophic Drivers of Scarabaeoidea Assemblages Along an Elevational Gradient in a Tropical Island Rainforest in China

**DOI:** 10.1002/ece3.73873

**Published:** 2026-06-19

**Authors:** Haoyan Sun, Yi Chen, Ruonan Hou, Lingbing Wu

**Affiliations:** ^1^ School of Tropical Agriculture and Forestry Hainan University Haikou China

**Keywords:** abundance, Bawangling, elevational gradient, top‐down effect, tropical mountain

## Abstract

Mountains serve as ideal natural laboratories for investigating biodiversity patterns along elevational gradients. This study examined α‐diversity (species richness and abundance) and β‐diversity (overall compositional dissimilarity and its turnover and nestedness) of Scarabaeoidea along an elevational gradient in a tropical rainforest, China. Scarabaeoidea assemblages were sampled continuously from early March to late November 2024 across 32 plots along an elevational gradient (371–1472 m a.s.l.) using ground and canopy traps. The aims were to evaluate the effects of potential drivers (temperature, humidity, litter biomass) and predator community (diversity and composition) on α‐ and β‐diversity. Scarabaeoidea species richness showed no significant relationship with elevation but increased with litter biomass. Scarabaeoidea abundance exhibited a pronounced mid‐elevational peak primarily driven by temperature, reflecting the strong thermal sensitivity of these ectotherms. Multisite β‐diversity was high (*β*_SOR_ = 0.893) and dominated by species turnover (*β*_SIM_ = 0.846). Pairwise β‐diversity (*β*_sor_) of predator assemblages was positively correlated with that of Scarabaeoidea assemblages, suggesting the potential trophic effects on community structure. Elevational distance was positively associated with species turnover (*β*_sim_) and *β*_sor_, but negatively associated with nestedness (*β*_sne_). Temperature distance remained a significant positive driver of turnover and β‐diversity and a negative driver of nestedness even after accounting for elevational distance. This study reveals that environmental filtering and trophic interactions jointly shape the assembly of Scarabaeoidea community in the Bawangling mountain. Our findings emphasize the importance of incorporating abundance and top‐down effects‐which may covary with environmental gradients‐when inferring the mechanisms structuring insect communities along elevational gradients.

## Graphical abstract

An important issue with diversity patterns along elevational gradients in mountain ecosystems is that far few studies have incorporated abundance and top‐down effects‐which may covary with environmental gradients‐when inferring the mechanisms structuring insect communities. Here, we tested the effects of potential drivers (temperature, humidity, litter biomass) and predator community (diversity and composition) on α‐diversity (species richness and abundance) and β‐diversity (overall compositional dissimilarity and its turnover and nestedness) of 32 Scarab beetles assemblages along an elevational gradient (371–1472 m a.s.l.) in a tropical rainforest, China. This study reveals that environmental filtering and trophic interactions jointly shape the assembly of Scarabaeoidea community in the Bawangling mountain. Our findings emphasize the importance of incorporating abundance and top‐down effects‐which may covary with environmental gradients‐when inferring the mechanisms structuring insect communities along elevational gradients.

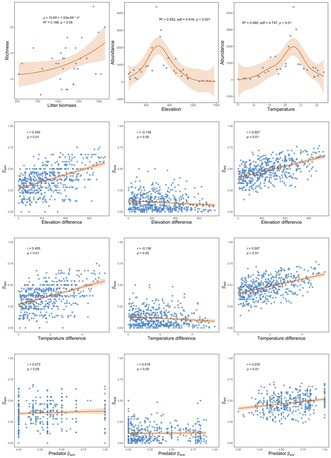

## 1 Introduction

Mountains are widely recognized as global biodiversity hotspots and provide exceptional ecosystems for elucidating patterns and drivers of elevational biodiversity across steep environmental gradients (Brehm et al. 2003; Graham et al. 2009; Noriega and Realpe 2018). Elevation incorporates sharp and spatially compressed differences in temperature, humidity, and vegetation structure (Rahbek 1995; Hodkinson 2005; Körner 2007; McCain 2007; Moctezuma et al. 2024), which jointly shape biodiversity distributions via physiological constraints, environmental filtering, and trophic interaction. These gradients are particularly pronounced in tropical regions, where reduced seasonal fluctuations constrain thermal tolerances, making mountain passes more effective barriers to dispersal and gene flow (Janzen 1967). As a result, tropical mountains often harbor extraordinary levels of endemism and exhibit significant compositional changes along elevational gradients.

Research on elevational diversity gradients has revealed a wide spectrum of α‐diversity patterns. McCain and Grytnes (2010) categorize elevational species richness patterns into four principal forms across ecosystems including a linear decline, a low‐elevation plateau followed by a sharp decrease, a plateau at low elevations with a mid‐elevational peak, and a unimodal mid‐elevational peak. Meanwhile, meta‐analyses of terrestrial vertebrates indicate that the prevalence of specific elevational richness patterns is strongly taxon dependent (McCain 2007; McCain and Grytnes 2010). For example, non‐volant small mammals typically show mid‐elevational richness peaks (McCain 2005), whereas bats exhibit more variable elevational patterns, with similar support for both monotonic and mid‐elevational peaks (McCain 2007). Birds and reptiles both exhibited all four common elevational species richness patterns; although these patterns were more evenly represented in birds, reptiles were characterized by a predominance of decreasing richness with elevation distribution (McCain and Grytnes 2010). Although comprehensive meta‐analytical syntheses remain lacking for insects, existing studies indicate that all major elevational species richness patterns occurred across different insect groups. Insect species richness can vary markedly along elevational gradients, with responses ranging from monotonic increases or decreases to unimodal patterns or the absence of a consistent elevational trend across taxa (Hodkinson 2005). Frequently evidenced hump‐shaped elevational richness patterns in insect groups such as geometrid moths (Brehm et al. 2003), epigaeic beetles (Werenkraut and Ruggiero 2014), and dung beetles (Escobar et al. 2005; da Silva et al. 2018) have generally been attributed to the combined effects of climatic and ecological factors, including intermediate thermal conditions, optimal productivity, and high habitat heterogeneity. In contrast, monotonic declines in Scarabaeidae richness have been primarily associated with physiological constraints and increasingly harsher environmental conditions at higher elevations (Hanski 1983; Hanski and Cambefort 1991; Lobo and Halffter 2000; Hodkinson 2005; Gebert et al. 2020).

Elevation gradients influence multiple dimensions of biodiversity, including species richness, abundance, and community composition. Yet, abundance trends with elevation have received comparatively less attention, even though evidence that elevational patterns of abundance and richness might arise from different environmental determinants (Werenkraut and Ruggiero 2014). For example, Werenkraut and Ruggiero (2014) suggested that the predominance of hump‐shaped elevational abundance patterns in epigaeic beetles across five temperate mountains in north‐western Argentina was primarily associated with variation in vegetation type, litter biomass and gravel cover. In contrast, β‐diversity and its spatial turnover and nestedness, have been increasingly employed to characterize compositional changes along elevational gradients, thereby yielding mechanistic insights into the underlying ecological processes underlying community assembly. For most terrestrial insects on mountains, β‐diversity typically exhibits an increasing trend with elevational distance and is predominantly driven by spatial turnover rather than nestedness (Hortal et al. 2011; Espinoza and Noriega 2018; da Silva et al. 2018). Such predominance of turnover is commonly attributed to deterministic species sorting across steep environmental gradients and restricted dispersal, while nestedness becomes more pronounced under conditions that promote the non‐random species loss (Baselga 2010). For example, studies on moths (Beck and Khen 2007) and dung beetles (da Silva et al. 2018) revealed that species turnover predominantly drives compositional variation with elevation, while nestedness reflects the selective loss of lowland taxa under cooler and less productive high‐elevation conditions (Baselga 2010; Hortal et al. 2011).

At a local scale, biodiversity patterns along elevational gradients result from the synergistic drivers associated with processes of environmental filtering, biotic interactions, and dispersal limitation. Mid‐elevation areas typically provide mesoclimates with moderate temperatures, relatively stable environments, and optimal productivity driven by energy and nutrient balance (Rahbek 1995; Waide et al. 1999). Furthermore, high environmental heterogeneity enhances habitat complexity and promotes species coexistence (Stein et al. 2014). In contrast, high‐altitude environments are characterized by lower temperatures, more severe climatic conditions (Rahbek 1995), and limited resources, which constrain energy flows and organism metabolism (Waide et al. 1999). These abiotic gradients also modulate the strength of biotic interactions, particularly predation. Predation has a major effect on community structure across trophic levels (Sandom et al. 2013; Chen et al. 2025). For instance, predators play a critical role in regulating herbivorous insect communities (Hairston et al. 1960; Paine 1980). However, to the best of our knowledge, whether such top‐down control interplays with ecological processes that shape community assembly of beetle assemblages in tropical montane ecosystems remains poorly understood.

The superfamily Scarabaeoidea (Coleoptera) offers an ideal group for exploring the mechanisms driving elevational diversity in insects. This group, which includes dung beetles, flower chafers, and rhinoceros beetles, plays essential ecological roles in nutrient cycling, decomposition, soil aeration, and secondary seed dispersal (Nichols et al. 2008). Their sensitivity to microclimate and habitat changes makes them reliable bioindicators of ecological integrity (Ritcher 1958; Hanski and Cambefort 1991; Noriega et al. 2021). Studies in both tropical and neotropical mountain ecosystems have demonstrated that Scarabaeoidea richness and abundance often peak at intermediate elevations, where favorable temperature and litter conditions promote their activity (Escobar et al. 2005; Moctezuma et al. 2016; da Silva et al. 2018).

In this study, we investigated 32 Scarabaeoidea assemblages from 371 to 1472 m a.s.l. to assess how Scarabaeoidea α‐ and β‐diversity changed along the elevational gradient in the Bawangling mountain within the National Park of Hainan Tropical Rainforest, and to test whether environmental filtering and trophic interactions determined these diversity patterns. To address this question, we aimed (1) to evaluate elevational patterns of α‐ (species richness and abundance) and β‐diversity (overall β‐diversity and its turnover and nestedness components), and (2) to determine whether environmental factors (temperature, humidity and litter biomass) and predator‐related variables (diversity and structure of predator community) acted as potential drivers of above α‐ and β‐diversity. We hypothesized that richness and abundance peak at mid‐elevations, where thermal and habitat conditions are optimal, and that β‐diversity increased with elevation distances due to environmental heterogeneity. This work aimed to reveal the determinants of tropical island montane Scarabaeoidea diversity and to provide scientific insights for Scarabaeoidea conservation in NPHTR.

## 2 Materials and Methods

### 2.1 Study Area

**FIGURE 1:**
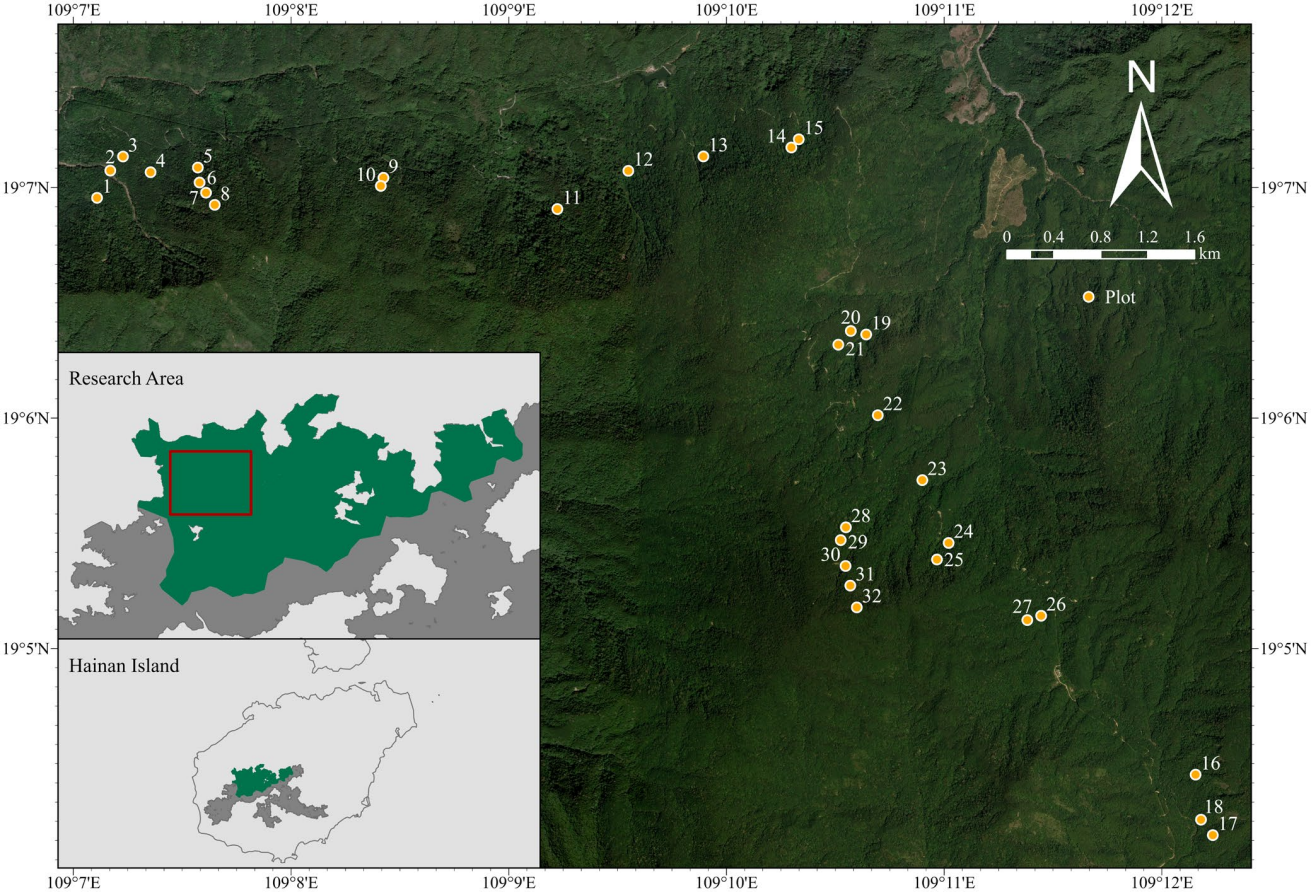
Location of the study area in the Bawangling mountain, Hainan Island, China. The inset shows the locations of all 32 sampling plots along the elevational gradient (371–1472 m a.s.l.) within the NPHTR. More information about the 32 plots can be seen in Table 1.

Hainan Island, the second largest island in China, separated from mainland China approximately 65 Ma (Liang 2018) and currently lies at the northern edge of the tropics. Bawangling mountain, located within the National Park of Hainan Tropical Rainforest (hereafter NPHTR), contains a continuous forest gradient from lowland to montane zones with minimal anthropogenic disturbance, making it an ideal system for investigating elevational diversity patterns. This study was conducted in the Bawangling rainforest (109°03′–109°17′ E, 18°57′–19°11′ N), located in the core area of NPHTR (Figure 1). Bawangling has a typical tropical monsoon climate characterized by mild seasonal variation but strong monsoonal circulation. The mean annual temperature is 21.3°C, with the warmest month averaging 22.8°C and the coldest month remaining above 13.5°C. Annual precipitation averages 1657 mm, with most rainfall concentrating between July and October and increasing with elevation. Bawangling mountain encompasses Mihouling (the third‐highest peak at 1654.8 m a.s.l.) and Heiling (the fourth‐highest peak at 1560.0 m a.s.l.). Vegetation composition varies markedly along the elevation gradient, transitioning from lowland deciduous monsoon forest and lowland rainforest below 800 m a.s.l., to montane rainforest at mid‐elevations, and to cloud forest above 1100 m a.s.l. (Long et al. 2011). The soil is primarily composed of Acrisols, developed from granite and sandstone at low altitudes, gradually transitioning to mountain red soil at higher altitudes (Hainan Forestry Department 2019). The complex topography and vegetation structure create diverse microhabitats, with temperature and humidity varying substantially along elevation. This pronounced environmental heterogeneity likely drives the diverse vertical distribution patterns and species turnover of Scarabaeoidea, reflecting their adaptation to specific habitat conditions within the tropical montane ecosystem.

### 2.2 Scarabaeoidea Assemblage Sampling

We established 32 sampling plots (20 m × 20 m, 400 m^2^ each) spaced at least 50 m apart along an elevational gradient (371 to 1472 m a.s.l.). In each plot, Scarabaeoidea assemblage was continuously sampled using nine ground traps and five canopy traps from early March to late November 2024. Ground traps were spaced approximately 5 m apart and filled with a mixture of ethanol and ethylene glycol (1:2 v/v). Each trap was covered with a plastic shield (25 cm in diameter) about 30 cm above the opening to prevent rainwater and leaf litter entry. Canopy traps were installed in the crowns of the five largest trees within each plot, positioned as close as possible to the main trunk at approximately 2 m in height. The trapping liquid was the same as used in ground traps. Beetle jelly was placed beneath plastic covers as supplemental bait.

To evaluate the potential drivers of the diversity of Scarabaeoidea assemblages, a set of environmental variables was recorded at each plot, including elevation, mean temperature, mean humidity, and litter biomass (Table 1). Temperature and humidity were continuously monitored using Temperature‐Moisture Sensor (TMS, TOMST Company, Czech Republic). Litter biomass was quantified using three 1 m × 1 m collection frames within each plot. Collected litter samples were dried at 80°C for 48 h, cooled to room temperature in a desiccator, and then weighed to determine dry biomass. To further test the role of predator presence in determining the diversity of Scarabaeoidea assemblages, two camera traps (Shenzhen Xinbai Information Technology Co. Ltd.) were installed at a height of 60 cm in each plot to monitor small vertebrate assemblages. Recorded vertebrate species were classified as potential predators of Scarabaeoidea according to their insectivorous diets, as verified using data from the China Species Library (https://species.sciencereading.cn/).

**TABLE 1:** Variables of study plots along the elevational gradient in the Bawangling mountain. Elevation (m), mean temperature (°C), mean humidity (%), litter biomass (g), predator species richness, and predator abundance were shown.

Plot	Elevation	Temperature	Humidity	Litter	Predator	
					Richness	Abundance
1	371	22.450	0.178	1461.70	20	274
2	389	22.064	0.230	1214.47	6	24
3	392	21.846	0.147	1042.74	2	16
4	439	21.924	0.152	1057.63	13	97
5	456	22.088	0.227	518.57	10	97
6	479	21.857	0.185	787.30	10	51
7	515	21.775	0.193	1273.59	0	0
8	536	21.392	0.234	804.50	5	23
9	595	20.959	0.212	1098.98	13	62
10	622	20.828	0.167	1187.95	15	123
11	674	20.224	0.184	1075.15	1	1
12	676	21.179	0.214	1220.81	9	34
13	730	20.521	0.218	1430.93	1	3
14	789	20.700	0.248	1092.31	12	92
15	778	20.347	0.256	1113.69	12	124
16	880	19.374	0.248	1005.39	18	266
17	822	20.053	0.275	1190.00	3	38
18	859	20.022	0.192	976.23	13	125
19	957	22.673	0.130	1291.04	9	54
20	977	19.078	0.232	1574.66	5	16
21	981	18.943	0.287	1350.76	11	91
22	1052	19.571	0.341	1241.14	7	15
23	1043	19.479	0.333	559.69	9	233
24	1073	18.579	0.243	928.15	11	137
25	1117	18.742	0.202	1036.03	12	85
26	1134	18.339	0.306	1495.55	1	1
27	1130	18.284	0.235	1026.29	2	2
28	1279	17.790	0.298	1116.49	3	6
29	1324	17.496	0.278	697.30	8	31
30	1387	17.369	0.289	1525.18	1	1
31	1422	—	—	1202.05	0	0
32	1472	17.029	0.257	1061.35	0	0

*Note:* ‘—’ indicates missing data due to instrument malfunction.

All collected specimens were preserved in 95% ethanol for subsequent taxonomic identification and are currently deposited at Hainan University. Species identification was performed using standard taxonomic keys and verified through image databases available from Global Biodiversity Information Facility (GBIF, www.gbif.org) and the Catalog of Life (www.catalogueoflife.org).

### 2.3 Data Analyses

Species with fewer than three individuals in the pooled dataset were excluded from analyses to reduce the potential influence of incidental occurrences, which may obscure the detection of niche‐based assembly patterns (Murray et al. 2017). Sampling completeness at each plot was assessed using sample coverage estimates calculated with the iNEXT package (Chao et al. 2014). To explore potential nonlinear relationships between diversity metrics and explanatory variables, generalized additive models (GAMs) were fitted using the mgcv package (Wood 2017) with the restricted maximum likelihood (REML) method. Predictors exhibiting approximately linear responses were analyzed using linear models to quantify their effects. The normality of residuals from all linear models was evaluated using the Shapiro–Wilk test. For abundance data, we also fitted generalized linear models (GLMs) with Poisson and negative binomial distributions to account for count‐data structure and potential overdispersion. To assess multicollinearity among potential drivers, we computed variance inflation factors (VIFs). When elevation was included in the full model, both elevation (VIF = 31.71) and temperature (VIF = 27.79) exhibited strong collinearity. After removing elevation, all remaining drivers showed acceptable VIF values below 10, confirming that multicollinearity was sufficiently reduced.

The corrected Akaike Information Criterion (AICc) was used to evaluate model fit. Subsequently, multi‐factor linear models were constructed to assess the combined effects of potential drivers on alpha diversity, and model averaging was performed for models with ΔAIC < 2 using the MuMIn package (Bartoń 2025) to improve the robustness of parameter estimates.

For β‐diversity, multisite (*β*_SOR_) and pairwise (*β*_sor_) Sørensen dissimilarity were partitioned into respective spatial turnover (*β*_SIM_, *β*_sim_) and nestedness (*β*_SNE_, *β*_sne_) components respectively using the betapart package (Baselga 2010). Mantel tests in the vegan package (Oksanen et al. 2025) were employed to assess the correlations between the distance matrices of potential drivers and assemblage dissimilarity matrices. Spearman rank correlation was applied because the data violated the normality assumption. As significant correlations were detected, distance between matrices of differences in elevation and temperature, as well as humidity (Table S1), partial Mantel tests were further performed to account for the confounding influence.

Approximate half of the Scarabaeoidea species collected were found as singletons or doubletons (47.8%), consistent with patterns frequently observed in insect community datasets (Colares et al. 2021). Therefore, to evaluate the robustness of our results, all analyses were repeated using the complete species dataset, without applying the previous abundance threshold of three individuals. All analyses were performed in R 4.5.1 (R Core Team 2025).

## 3 Results

### 3.1 Sampling Completeness and Taxonomic Composition

The sampling coverage for each study plot exceeded 0.85 (0.859–0.999) with the accumulation curve approaching its asymptote, indicating that our sampling completeness for each plot was reliable (Figure S1). In total, 26,665 Scarabaeoidea individuals from 48 species (25 genera, 8 subfamilies) were collected (Table S2). *Onthophagus lunatus* (Harold 1868) was a super‐dominant species, accounting for 86.99% of the total individuals. The majority of the species were from Rutelinae (13 species), followed by Scarabaeinae (12), Lucaninae (7), Cetoniinae (6), Melolonthinae (4), Geotrupidae (2), Hybosoridae (2), and Dynastinae (1). A total of 34 insectivorous predator species were recorded, comprising 23 bird species and 11 mammal species (Table S3). An additional 44 species, each represented by fewer than three individuals in the pooled dataset, were provided in Table S2.

### 3.2 Species Richness Pattern Along the Elevational Gradient

**FIGURE 2:**
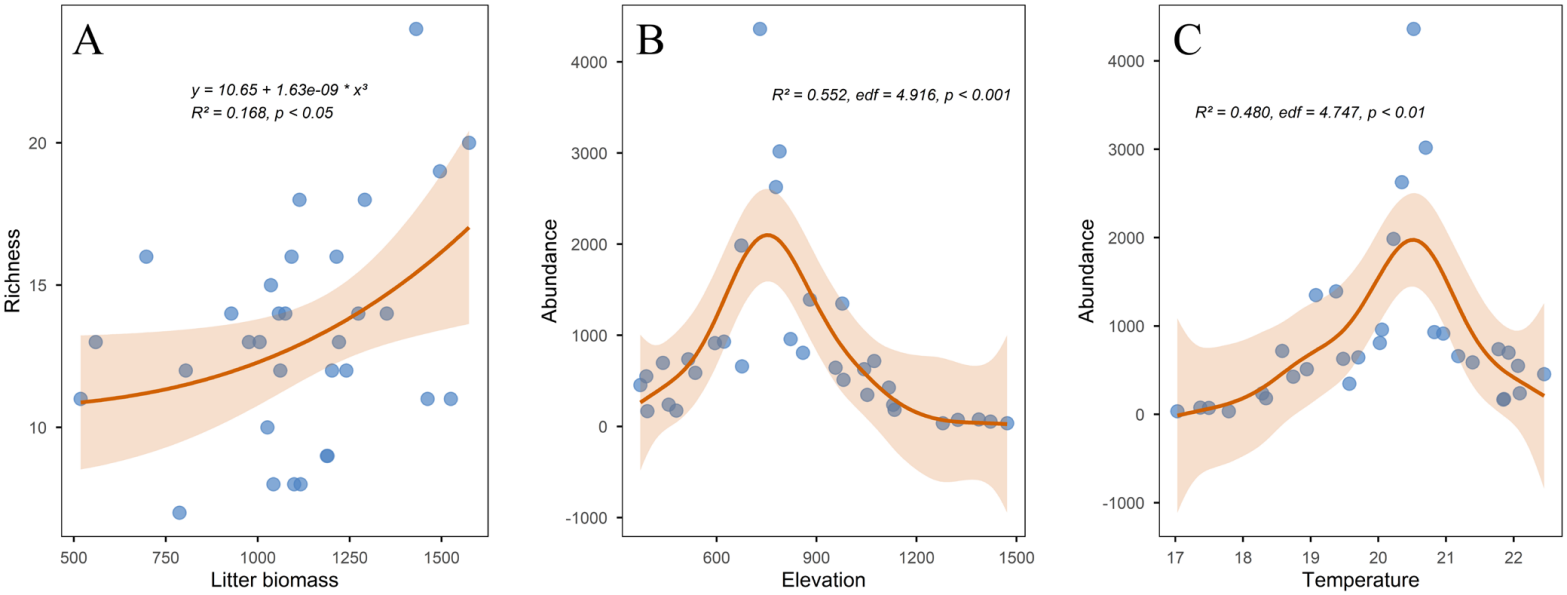
Nonlinear relationships between potential drivers and Scarabaeoidea species richness (A) and abundance (B, C) along the elevational gradient within the Bawangling mountain. The shaded area indicates the 95% confidence interval.

No significant linear or nonlinear relationship was found between elevation and Scarabaeoidea species richness (all *p* > 0.05, Figure S2). In contrast, species richness showed a significant positive relationship with litter biomass, with the cubic term providing the best fit (*R* ^2^ = 0.168, *p* = 0.020; Figure 2A and Figure S3). The model fit for Scarabaeoidea species richness according to AICc showed that the top‐ranked model was the one containing only litter biomass. The multi‐factor model with delta AICc < 2 was “species richness ~ litter + humidity” (Table 2). However, neither the coefficient for litter (*p* = 0.053) nor for humidity (*p* = 0.427) in this model was statistically significant, and the overall model was not significant (*p* = 0.111). Analyses conducted without the three‐individual yielded consistent results. The Supporting Information compared results generated with and without applying an abundance threshold of three individuals (Tables S4 and S5).

**TABLE 2:** Model selection results from linear models of relationships between potential drivers and Scarabaeoidea species richness along the elevational gradient within the Bawangling mountain based on corrected Akaike Information Criterion (AICc).

Model	AICc	ΔAICc	Weight
Litter	174.1	0.00	0.412
Null model	175.8	1.69	0.177
Litter + humidity	176.0	1.94	0.156

### 3.3 Abundance Pattern Along the Elevational Gradient

The preliminary GLMs with Poisson showed substantial overdispersion, resulting in inflated significance levels. GLMs with negative binomial distribution reduced overdispersion but showed relatively low explanatory power (Tables S6, S7 and Figure S4, low *R* ^2^ values). Therefore, GAMs were considered more appropriate and were used for inference. Scarabaeoidea abundance exhibited a significant hump‐shaped trend with elevation and temperature, with a peak at 730 m a.s.l. and 20.5°C (Figure 2B,C). No significant linear or nonlinear relationship was detected with predator species richness, predator abundance, and humidity (all *p* > 0.05).

Model selection based on AICc indicated that the top‐ranked model included only temperature (Table 3), which had a significant positive effect on Scarabaeoidea abundance from simple linear model (Figure 2). Other competing models that incorporated litter biomass, predator abundance, or predator richness (all with ΔAICc < 2) did not improve model performance, and none of these additional drivers showed significant effects from multilinear models (all *p* > 0.05). Analyses conducted without the three‐individual yielded consistent results. The Supporting Information compared results generated with and without applying abundance threshold of three individuals (Tables S8 and S9).

**TABLE 3:** Model selection results for generalized additive models (GAMs) of relationships between potential drivers and Scarabaeoidea abundance and potential drivers based on corrected Akaike information criterion (AICc).

Model	AICc	ΔAICc	Weight
Temperature	511.1	0.00	0.153
Temperature + litter	512.2	1.14	0.087
Temperature + humidity	512.9	1.66	0.067

### 3.4 β‐Diversity Pattern Along the Elevational Gradient

Multisite β‐diversity was high (*β*_SOR_ = 0.893) and overwhelmingly driven by species turnover (*β*_SIM_ = 0.846), which contributed 94.78% to the total. This suggests the dominance of species turnover and the negligible importance of nestedness among Scarabaeoidea assemblages.

Both differences in elevation and temperature exhibited significant positive correlations with *β*_sim_ and *β*_sor_ but negative correlations with *β*_sne_. Differences in humidity also exhibited significant positive correlations with *β*_sim_ and *β*_sor_ (Table 4 and Figure S5). *β*_sor_ of predators displayed a significant positive correlation with Scarabaeoidea *β*_sor_, whereas it showed no significant association with *β*_sim_ and *β*_sne_. Litter showed no significant association with any dissimilarities.

**TABLE 4:** Mantel and partial Mantel (in parentheses) correlations between assemblage dissimilarity matrices (*β*_sim_, *β*_sne_, and *β*_sor_) and distance matrices of potential drivers.

Predictor	*β*_sim_	*β*_sne_	*β*_sor_
Elevation	0.455***	−0.136***	0.507***
Temperature	0.427*** (0.299**)	−0.132*** (−0.132*)	0.464*** (0.266**)
Humidity	0.155* (0.018)	−0.064 (−0.021)	0.160* (0.013)
Litter	−0.111	0.058	−0.086
β of predators	0.073	0.019	0.235**
Predator abundance	0.022	−0.109	−0.073

* *p* < 0.05.

** *p* < 0.01.

*** *p* < 0.001.

Partial Mantel tests showed that differences in temperature remained a strongly significant driver of *β*_sim_, *β*_sor_, and *β*_sne_ independent of elevation. However, the significant associations between humidity and dissimilarities became non‐significant after controlling for elevation (Table 4). Analyses conducted without the three individuals yielded consistent results. The inclusion of rare species did not alter the main conclusions, thereby strengthening the robustness of our findings (Table S10).

## 4 Discussion

This study revealed contrasting elevational patterns in Scarabaeoidea richness and abundance along the tropical montane gradient of Bawangling mountain, Hainan Island, China. Abundance showed a pronounced hump‐shaped pattern, with a peak at 730 m a.s.l. (Figure 2B,C), whereas species richness exhibited no apparent trend across the gradient (371–1472 m a.s.l.). Temperature was the strongest predictor of abundance, while litter biomass best explained the variation in richness. β‐diversity was dominated by species turnover, indicating substantial species replacement among elevations. This turnover‐dominated pattern was jointly explained by temperature and predator community composition, suggesting that both abiotic filtering and biotic interactions contribute to Scarabaeoidea community structure along the elevational gradient. To our knowledge, this study provides the first assessment of elevational patterns and potential mechanisms shaping Scarabaeoidea richness, abundance and community structure within the NPHTR.

### 4.1 Patterns and Drivers of Species Richness and Abundance Along the Elevational Gradient

Contrary to our prediction, Scarabaeoidea species richness in the Bawangling mountain showed no significant linear or nonlinear relationship with elevation. Such nonsignificant elevational trend has been reported in other tropical montane Scarabaeoidea assemblages (e.g., Mongyeh et al. 2018). The absence of a clear elevational pattern in Scarabaeoidea richness observed in our study might be attributable to the combined effects of multiple determinants that shaped Scarabaeoidea occurrence, such as dung and carrion resource availability (Hanski and Cambefort 1991; Gebert et al. 2020), vegetation type (Werenkraut and Ruggiero 2014), and primary productivity (Werenkraut and Ruggiero 2014; da Silva et al. 2018). In this study, litter biomass was identified as the sole driver positively associated with Scarabaeoidea species richness (Figure 2A). Litter constitutes a critical habitat complexity by simultaneously providing shelter and breeding substrates, which also buffering temperature fluctuations and retaining soil moisture. These structural and thermal buffering properties might create favorable conditions that support both adult activities and, more critically, promote successful larval development (Lindman et al. 2022).

Consistent with our hypothesis, Scarabaeoidea abundance exhibited a pronounced hump‐shaped elevation pattern, reaching its maximum at mid‐elevations (Figure 2B). Similar mid‐elevation peaks in Scarabaeoidea abundance along montone gradients have been reported in the Neotropics (da Silva et al. 2018; Joaqui et al. 2021; Holzmann et al. 2026), Afrotropics (Gebert et al. 2020), and Southeast Asia (Hanski and Cambefort 1991). These studies attributed this mid‐elevational peak to the convergence with distinct environmental adaptations, metabolism requirements, and biogeographic origins at intermediate elevations. Abundance reflects local population performance and persistence in a specific habitat (Cadotte and Tucker 2017). In our study, mid‐elevation habitats in Bawangling rainforest are characterized by moderate temperature and humidity, which are expected to promote Scarabaeoidea reproduction and feeding. Such intermediate environments might reduce physiological stress, providing favorable conditions for species persistence and activity (Körner 2007). Mid‐elevational habitats often provide thermally and hydrologically moderate conditions that enhance activity, reproduction, and survival of Scarabaeoidea (Janzen 1967). Thus, elevated abundance at mid‐elevations may reflect physiological optimization under favorable microclimatic regimes, even when species richness shows no clear elevational trend due to other constraints.

Our finding confirmed that the abundance of Scarabaeoidea was primarily regulated by temperature (Figure 2C). This pronounced thermal sensitivity is consistent with evidence from Neotropical and Asian montane systems, where microclimatic stability was recognized as a key determinant of Scarabaeoidea diversity and distribution (Espinoza and Noriega 2018; da Silva et al. 2018). Intermediate elevations may provide more stable environmental and climatic conditions, whereas lower and higher elevations may be subject to greater variability and more frequent exposure to harsh events (da Silva et al. 2018). The dominant role of abiotic filtering in shaping Scarabaeoidea abundance along elevational gradients has been widely documented in previous studies (*e.g*., da Silva et al. 2018; Gebert et al. 2020). Our result provides evidence that abundance represents a meaning covariance between the persistence of a biological group and specific environmental qualities. For Scarabaeoidea, this temperature dependence likely reflects physiological thresholds governing flight activity, feeding, and reproductive behavior, all of which are strongly constrained by ambient temperature (Hanski and Cambefort 1991). Specifically, physiological thresholds linked to temperature frequently constrain species survival, reproduction, and dispersal capacity in montane environments (Menéndez et al. 2014). For ectotherms, thermoregulation toward preferred body temperatures can buffer the effects of ambient temperature on metabolic rates and performance, provided that environmental conditions are warm enough for activity (Huey et al. 2003). Our finding further support the predominant role of temperature in shaping abundance pattern of ectothermic taxa along elevational gradients (Buckley et al. 2012). However, as our analysis relied on mean temperature, it may not fully capture the temporal variability that could influence Scarabaeoidea abundance across the sampling period.

The dominance of temperature and litter availability over other environmental variables suggests that thermal and habitat filtering jointly regulated α‐diversity of Scarabaeoidea assemblages along the elevational gradient within the Bawangling mountain. This pattern is consistent with observations reported for multiple beetles' taxa across montane ecosystems (Colares et al. 2021). The environmental filtering paradigm is further reinforced by evidence that high‐elevation conditions frequently constrain insect activity via physiological thresholds (Hodkinson 2005). Our findings support the broader environmental filtering framework (Cadotte and Tucker 2017), whereby abiotic constraints and habitat structure are likely to promote environmental filtering that interacts to determine community composition and population outcome.

### 4.2 Patterns and Drivers of β‐Diversity Along the Elevational Gradient

Our study reveals that the spatial structuring of Scarabaeoidea assemblages along the Bawangling elevational gradient is overwhelmingly driven by species turnover (*β*_SIM_), with a negligible contribution from nestedness (*β*_SNE_). This turnover‐dominated pattern indicates that conditions at different elevations create unique assemblages rather than depauperate subsets of lower‐elevation faunas (Baselga 2010).

We observed that both β‐diversity (*β*_sor_) and its turnover (*β*_sim_) were positively correlated with differences in elevation and temperature (Table 4). This is consistent with the process of environmental filtering, where changes in abiotic conditions across space select for species with specific physiological tolerances, leading to increased spatial species turnover with differences in elevation and temperature. By contrast, the negative correlation with nestedness (*β*_sne_) further indicates an ordered loss of species with differences in elevation and temperature. Even after controlling for elevation, temperature independently explained turnover, emphasizing the importance of fine‐scale thermal variation over simply elevational position. The high rate of species turnover observed in our study suggests that the temperature gradient acts as a series of physiological hurdles, filtering species according to their thermal tolerances and leading to the replacement of taxa along elevation.

Our result that predator β‐diversity (*β*_sor_) was positively associated with the β‐diversity (*β*_sor_) of Scarabaeoidea assemblages (Table 4) highlights the close coupling between predator and prey communities along the elevational gradient. Predator diversity and community structure are not only influenced by bottom‐up effects from prey availability and diversity but are also drivers of top‐down control (Leles et al. 2017). From a top‐down perspective, greater predator diversity, including diversity of hunting strategies and body‐size variation, can promote prey diversification or coexistence through differential predation pressure (Hairston et al. 1960; Apfelbach et al. 2005). Our results therefore support the view that predator–prey interactions play an integral role in shaping community differentiation along environmental gradients (Hairston et al. 1960).

## 5 Conclusion

This study elucidated the effects of elevation, temperature, humidity, litter biomass, and predator assemblages on the diversity patterns and community structure of Scarabaeoidea assemblages along an elevational gradient within the National Park of Hainan Tropical Rainforest on Hainan Island, China. Although Scarabaeoidea species richness exhibited no clear elevational trend, abundance peaked at mid‐elevations. Temperature emerged as the primary environmental determinant of abundance, underscoring the thermal sensitivity of ectotherms and the central role of temperature in regulating their population dynamics. Community dissimilarity was overwhelmingly driven by species turnover rather than nestedness, implying that multiple elevational bands contribute uniquely to Scarabaeoidea γ‐diversity and therefore merit conservation attention within the Bawangling mountain. Temperature and predator composition jointly explained this turnover‐dominated β‐diversity pattern along the elevational gradient. The strong temperature dependence observed in Scarabaeoidea diversity in our study further suggests that these assemblages may be highly vulnerable to climate change. Our findings demonstrate that both abiotic filters such as elevation, temperature, litter biomass, and biotic interactions, including trophic dynamics, jointly shape the Scarabaeoidea communities in this tropical island mountains. Our study stress the importance of integrating abundance and top‐down effects, which may covary with environmental gradients, to more accurately infer the mechanisms structuring communities along elevational gradients.

## Author Contributions

**Haoyan Sun:** data curation (equal), formal analysis (equal), investigation (equal), methodology (equal), software (equal), validation (equal), visualization (equal), writing – original draft (equal), writing – review and editing (equal). **Yi Chen:** investigation (equal), validation (equal), visualization (equal). **Ruonan Hou:** validation (equal), visualization (equal), writing – review and editing (equal). **Lingbing Wu:** conceptualization (lead), data curation (lead), formal analysis (lead), funding acquisition (lead), investigation (equal), methodology (lead), project administration (lead), resources (lead), software (lead), supervision (lead), validation (lead), visualization (lead), writing – original draft (equal), writing – review and editing (equal).

## Funding

This work was supported by Hainan Provincial Natural Science Foundation of China (322MS023), the National Natural Science Foundation of China (31901215), the China Postdoctoral Science Foundation (2017M622383), the International Postdoctoral Exchange Fellowship Program (PC2018027).

## Conflicts of Interest

The authors declare no conflicts of interest.

## Supporting information


**Table S1:** Spearman rank correlations among potential drivers. *p < 0.05; **p < 0.01; ***p < 0.001.
**Figure S1:** Sampling completeness (SC) of Scarabaeoidea assemblages for 32 plots in the Bawangling mountain.
**Table S2:** List of Scarabaeoidea species and their total individuals collected from 32 sampling plots along the elevational gradient in the Bawangling mountain.
**Table S3:** List of predator species monitored from 32 sampling plots along the elevational gradient in the Bawangling mountain.
**Figure S2:** Relationships between Scarabaeoidea species richness and litter biomass.
**Table S4:** The comparison of results from simple linear models for each potential driver and Scarabaeoidea species richness along the elevational gradient in the Bawangling mountain with and without applying abundance threshold of three individuals.
**Table S5:** Model selection results from linear models of relationships between potential drivers and Scarabaeoidea species richness along the elevational gradient in the Bawangling mountain based on corrected Akaike Information Criterion (AICc) without applying abundance threshold of three individuals.
**Table S6:** Results of Poisson and negative binomial models for individual predictors of Scarabaeoidea abundance with abundance threshold of three individuals.
**Figure S3:** Relationships between Scarabaeoidea abundance and potential drivers (using negative binomial distribution).
**Table S7:** The comparison of results from generalized additive models (GAMs) of relationships between each potential driver and Scarabaeoidea abundance along the elevational gradient in the Bawangling mountain based on corrected Akaike Information Criterion (AICc) with and without applying abundance threshold of three individuals. The estimated degree of freedom (edf) of each model was provided.
**Table S8:** Model selection results from generalized additive models (GAMs) of relationships between potential drivers and Scarabaeoidea abundance based on corrected Akaike Information Criterion (AICc) without applying abundance threshold of three individuals.
**Figure S4:** Mantel tests between Scarabaeoidea *β* and elevation, temperature and predator *β*.
**Table S9:** Mantel correlations between assemblage dissimilarity matrices (*β*_sim_, *β*_sne_, and *β*_sor_) and distance matrices of potential drivers without applying abundance threshold of three individuals. *p < 0.05; **p < 0.01; ***p < 0.001.

## Data Availability

All the required data are uploaded as Supporting Information.
