# Supplementary material for: Turnover‐Dominated β‐Diversity and Its Temperature and Trophic Drivers of Scarabaeoidea Assemblages Along an Elevational Gradient in a Tropical Island Rainforest in China
Source: Ecol Evol. 2026 Jun 19;16(6):e73873. doi: 10.1002/ece3.73873 (PMC13280470; doi:10.1002/ece3.73873)
Supplement: Supplementary file 1 — Table S1: Spearman rank correlations among potential drivers. *p < 0.05; **p < 0.01; ***p < 0.001. Figure S1: Sampling completeness (SC) of Scarabaeoidea assemblages for 32 plots in the Bawangling mountain. Table S2: List of Scarabaeoidea species and their total individuals collected from 32 sampling plots along the elevational gradient in the Bawangling mountain. Table S3: List of predator species monitored from 32 sampling plots along the elevational gradient in the Bawangling mountain. Figure S2: Relationships between Scarabaeoidea species richness and litter biomass. Table S4: The comparison of results from simple linear models for each potential driver and Scarabaeoidea species richness along the elevational gradient in the Bawangling mountain with and without applying abundance threshold of three individuals. Table S5: Model selection results from linear models of relationships between potential drivers and Scarabaeoidea species richness along the elevational gradient in the Bawangling mountain based on corrected Akaike Information Criterion (AICc) without applying abundance threshold of three individuals. Table S6: Results of Poisson and negative binomial models for individual predictors of Scarabaeoidea abundance with abundance threshold of three individuals. Figure S3: Relationships between Scarabaeoidea abundance and potential drivers (using negative binomial distribution). Table S7: The comparison of results from generalized additive models (GAMs) of relationships between each potential driver and Scarabaeoidea abundance along the elevational gradient in the Bawangling mountain based on corrected Akaike Information Criterion (AICc) with and without applying abundance threshold of three individuals. The estimated degree of freedom (edf) of each model was provided. Table S8: Model selection results from generalized additive models (GAMs) of relationships between potential drivers and Scarabaeoidea abundance based on corrected Akaike Information Criterion (A [file ECE3-16-e73873-s001.docx]

Table S1 Spearman rank correlations between potential drivers. *, p < 0.05; **, p < 0.01; ***, p < 0.001.

|  | Elevation | Temperature | Humidity | Litter biomass | Predator richness |
| --- | --- | --- | --- | --- | --- |
| Temperature | 0.954^***^ |  |  |  |  |
| Humidity | 0.333^***^ | 0.250^**^ |  |  |  |
| Litter biomass | 0.028 | 0.054 | 0.019 |  |  |
| Predator richness | 0.096 | 0.128^*^ | 0.003 | 0.024 |  |
| Predator abundance | 0.031 | 0.060 | 0.057 | 0.110 | 0.782^***^ |





FIGURE S1 Sampling completeness (SC) of Scarabaeoidea assemblages for 32 plots in the Bawangling mountain. The shaded area indicates the 95% confidence interval.

Table S2 List of Scarabaeoidea species and their total individuals (Ind) collected from 32 sampling plots along the elevational gradient in the Bawangling mountain.

|  | Family | Subfamily | Species | Ind |
| --- | --- | --- | --- | --- |
| 1 | Geotrupidae | Bolboceratinae | *Bolbochromus laetus* (Westwood, 1852) | 3 |
| 2 | Geotrupidae | Geotrupinae | *Phelotrupes bolm* (Král, Malý & Schneider, 2001) | 5 |
| 3 | Hybosoridae | Hybosorinae | *Phaeochroops* sp. 1 | 1 |
| 4 | Hybosoridae | Hybosorinae | *Phaeochroops taiwanus* (Nomura, 1973) | 97 |
| 5 | Hybosoridae | Hybosorinae | *Phaeochrous hainanensis* (Zhang, 1990) | 8 |
| 6 | Lucanidae | Lucaninae | *Epidorcus tonkinensis* (Pouillaude, 1913) | 3 |
| 7 | Lucanidae | Lucaninae | *Figulus binodulus* (Waterhouse, 1872) | 1 |
| 8 | Lucanidae | Lucaninae | *Figulus* sp. 1 | 3 |
| 9 | Lucanidae | Lucanidae | *Hexarthrius vitalisi* (Didier, 1925) | 1 |
| 10 | Lucanidae | Lucanidae | *Lucanus hermani* (Lisle, 1973) | 1 |
| 11 | Lucanidae | Lucaninae | *Neolucanus nitidus* (Saunders, 1854) | 3 |
| 12 | Lucanidae | Lucaninae | *Neolucanus parryi* (Leuthner, 1885) | 17 |
| 13 | Lucanidae | Lucaninae | *Neolucanus sinicus* (Saunders, 1854) | 4 |
| 14 | Lucanidae | Lucaninae | *Neolucanus tao* (Kriesche, 1935) | 6 |
| 15 | Lucanidae | Lucaninae | *Odontolabis cuvera* (Hope, 1842) | 3 |
| 16 | Lucanidae | Lucaninae | *Odontolabis platynota* (Hope, 1845) | 4 |
| 17 | Lucaninae | Lucaninae | *Odontolabis siva* (Hope, 1845) | 1 |
| 18 | Lucaninae | Lucaninae | *Velutinodorcus velutinus* (Maes, 1992) | 1 |
| 19 | Passalidae | Macrolininae | *Aceraius grandis* (Burmeister, 1847) | 1 |
| 20 | Scarabaeidae | Cetoniinae | Cetoniinae sp. 1 | 4 |
| 21 | Scarabaeidae | Cetoniinae | Cetoniinae sp. 2 | 1 |
| 22 | Scarabaeidae | Cetoniinae | *Clinterocera davidis* (Fairmaire, 1878) | 1 |
| 23 | Scarabaeidae | Cetoniinae | *Cymophorus pulchellus* (Arrow, 1910) | 2 |
| 24 | Scarabaeidae | Cetoniinae | *Cymophorus* sp. 1 | 1 |
| 25 | Scarabaeidae | Cetoniinae | *Euselates furcata* (Kraatz, 1893) | 1 |
| 26 | Scarabaeidae | Cetoniinae | *Euselates ornata* (Saunders, 1852) | 1 |
| 27 | Scarabaeidae | Cetoniinae | *Euselates quadrilineata* (Hope, 1831) | 1 |
| 28 | Scarabaeidae | Cetoniinae | *Glycyphana horsfieldi* (Hope, 1831) | 3 |
| 29 | Scarabaeidae | Cetoniinae | *Glycyphana* sp. 1 | 1 |
| 30 | Scarabaeidae | Cetoniinae | *Paratrichius rufescens* (Ma, 1990) | 2 |
| 31 | Scarabaeidae | Cetoniinae | *Protaetia* sp. 1 | 2 |
| 32 | Scarabaeidae | Cetoniinae | *Rhomborhina resplendens* (Swartz, 1817) | 1 |
| 33 | Scarabaeidae | Cetoniinae | *Rhomborhina splendida* (Moser, 1913) | 2 |
| 34 | Scarabaeidae | Cetoniinae | *Rhomborhina unicolor* (Motschulsky, 1861) | 3 |
| 35 | Scarabaeidae | Cetoniinae | *Trichius bifasciatus* (Moser, 1901) | 1 |
| 36 | Scarabaeidae | Cetoniinae | *Valgus* sp. 1 | 56 |
| 37 | Scarabaeidae | Cetoniinae | *Valgus* sp. 2 | 11 |
| 38 | Scarabaeidae | Cetoniinae | *Valgus* sp. 3 | 2 |
| 39 | Scarabaeidae | Cetoniinae | *Valgus* sp. 4 | 4 |
| 40 | Scarabaeidae | Dynastinae | *Trichogomphus mongol* (Arrow, 1908) | 21 |
| 41 | Scarabaeidae | Melolonthinae | *Apogonia pilifera* (Moser, 1913) | 1 |
| 42 | Scarabaeidae | Melolonthinae | *Apogonia* sp. 1 | 3 |
| 43 | Scarabaeidae | Melolonthinae | *Cyphochilus apicalis* (Waterhouse, 1867) | 1 |
| 44 | Scarabaeidae | Melolonthinae | *Eotrichia hainanensis* (Zhang, 1964) | 1 |
| 45 | Scarabaeidae | Melolonthinae | *Exolontha serrulata* (Gyllenhal, 1817) | 9 |
| 46 | Scarabaeidae | Melolonthinae | *Holotrichia lata* (Brenske, 1892) | 19 |
| 47 | Scarabaeidae | Melolonthinae | *Holotrichia sinensis* (Hope, 1842) | 17 |
| 48 | Scarabaeidae | Melolonthinae | *Lepidiota stigma* (Fabricius, 1801) | 1 |
| 49 | Scarabaeidae | Rutelinae | *Adoretus maniculus* (Ohaus, 1914) | 1 |
| 50 | Scarabaeidae | Rutelinae | *Adoretus sinicus* (Burmeister, 1855) | 11 |
| 51 | Scarabaeidae | Rutelinae | *Anomala albopilosa* (Hope, 1839) | 6 |
| 52 | Scarabaeidae | Rutelinae | *Anomala antiqua* (Gyllenhal, 1817) | 2 |
| 53 | Scarabaeidae | Rutelinae | *Anomala aulax* (Wiedemann, 1823) | 34 |
| 54 | Scarabaeidae | Rutelinae | *Anomala australis* (Linnaeus, 2002) | 1 |
| 55 | Scarabaeidae | Rutelinae | *Anomala barbellate* (Linnaeus, 1996) | 2 |
| 56 | Scarabaeidae | Rutelinae | *Anomala edentula* (Ohaus, 1925) | 17 |
| 57 | Scarabaeidae | Rutelinae | *Anomala ignipes* (Linnaeus, 1996) | 1 |
| 58 | Scarabaeidae | Rutelinae | *Anomala obliquisulcata* (Linnaeus, 2002) | 2 |
| 59 | Scarabaeidae | Rutelinae | *Anomala profundisulca* (Linnaeus, 2002) | 4 |
| 60 | Scarabaeidae | Rutelinae | *Anomala russiventris* (Fairmaire, 1893) | 24 |
| 61 | Scarabaeidae | Rutelinae | *Anomala spiloptera* (Burmeister, 1855) | 9 |
| 62 | Scarabaeidae | Rutelinae | *Anomala virens* (Linnaeus, 1996) | 1 |
| 63 | Scarabaeidae | Rutelinae | *Maladera ovatula* (Fairmaire, 1891) | 8 |
| 64 | Scarabaeidae | Rutelinae | *Mimela chinensis* (Kirby, 1823) | 3 |
| 65 | Scarabaeidae | Rutelinae | *Mimela dentifera* (Linnaeus, 1990) | 151 |
| 66 | Scarabaeidae | Rutelinae | *Mimela excisipes* (Reitter, 1903) | 2 |
| 67 | Scarabaeidae | Rutelinae | *Mimela nubeculata* (Linnaeus, 1990) | 40 |
| 68 | Scarabaeidae | Rutelinae | *Mimela seminigra* (Ohaus, 1908) | 1 |
| 69 | Scarabaeidae | Rutelinae | *Mimela* sp. 1 | 1 |
| 70 | Scarabaeidae | Rutelinae | *Mimela* sp. 2 | 1 |
| 71 | Scarabaeidae | Rutelinae | *Mimela specularis* (Ohaus, 1902) | 31 |
| 72 | Scarabaeidae | Rutelinae | *Mimela sulcatula* (Ohaus, 1915) | 15 |
| 73 | Scarabaeidae | Scarabaeinae | *Catharsius sinicus* (Hope, 1837) | 1 |
| 74 | Scarabaeidae | Scarabaeinae | *Copris confucius* (Harold, 1877) | 30 |
| 75 | Scarabaeidae | Scarabaeinae | *Copris* sp. 1 | 125 |
| 76 | Scarabaeidae | Scarabaeinae | *Copris* sp. 2 | 6 |
| 77 | Scarabaeidae | Scarabaeinae | *Onitieellus cinctus* (Fabricius, 1775) | 2 |
| 78 | Scarabaeidae | Scarabaeinae | *Onitis falcatus* (Wulfen, 1786) | 27 |
| 79 | Scarabaeidae | Scarabaeinae | *Onitis spinipes* (Drury, 1770) | 2 |
| 80 | Scarabaeidae | Scarabaeinae | *Onthophagus basicruentatus* (Latreille, 1802) | 1970 |
| 81 | Scarabaeidae | Scarabaeinae | *Onthophagus lunatus* (Harold, 1868) | 23197 |
| 82 | Scarabaeidae | Scarabaeinae | *Onthophagus rectecornutus* (Lansberge, 1883) | 1 |
| 83 | Scarabaeidae | Scarabaeinae | *Onthophagus sagittarius* (Fabricius, 1775) | 4 |
| 84 | Scarabaeidae | Scarabaeinae | *Onthophagus seniculus* (Fabricius, 1781) | 2 |
| 85 | Scarabaeidae | Scarabaeinae | *Onthophagus* sp. 1 | 358 |
| 86 | Scarabaeidae | Scarabaeinae | *Onthophagus* sp. 2 | 40 |
| 87 | Scarabaeidae | Scarabaeinae | *Onthophagus* sp. 3 | 2 |
| 88 | Scarabaeidae | Scarabaeinae | *Onthophagus* sp. 4 | 1 |
| 89 | Scarabaeidae | Scarabaeinae | *Onthophagus* sp. 5 | 2 |
| 90 | Scarabaeidae | Scarabaeinae | *Onthophagus tricornis* (Waldheim, 1844) | 13 |
| 91 | Scarabaeidae | Scarabaeinae | *Onychothecus* sp. 1 | 5 |
| 92 | Scarabaeidae | Scarabaeinae | *Paragymnopleurus melanarius* (Harold, 1867) | 234 |

Table S3 List of predator species monitored from 32 sampling plots along the elevational gradient in the Bawangling mountain.

| Class | Order | Family | Species |
| --- | --- | --- | --- |
| Aves | Artiodactyla | Suidae | *Sus scrofa* (Linnaeus, 1758) |
| Aves | Carnivora | Mustelidae | *Melogale moschata* (Gray, 1831) |
| Aves | Carnivora | Viverridae | *Paradoxurus hermaphroditus* (Pallas, 1777) |
| Aves | Columbiformes | Columbidae | *Chalcophaps indica* (Linnaeus, 1758) |
| Aves | Eulipotyphla | Soricidae | *Suncus murinus* (Linnaeus, 1766) |
| Aves | Galliformes | Phasianidae | *Arborophila ardens* (Styan, 1892) |
| Aves | Galliformes | Phasianidae | *Gallus gallus* (Linnaeus, 1758) |
| Aves | Galliformes | Phasianidae | *Lophura nycthemera* (Linnaeus, 1758) |
| Aves | Passeriformes | Corvidae | *Cissa hypoleuca* (Salvadori & Giglioli, 1885) |
| Aves | Passeriformes | Dicruridae | *Dicrurus paradiseus* (Linnaeus, 1766) |
| Aves | Passeriformes | Emberizidae | *Emberiza aureola* (Pallas, 1773) |
| Aves | Passeriformes | Leiothrichidae | *Alcippe hueti* (David, 1874) |
| Aves | Passeriformes | Leiothrichidae | *Garrulax castanotis* (Ogilvie-Grant, 1899) |
| Aves | Passeriformes | Leiothrichidae | *Garrulax monileger* (Hodgson, 1836) |
| Aves | Passeriformes | Leiothrichidae | *Pterorhinus pectoralis* (Gould, 1836) |
| Aves | Passeriformes | Leiothrichidae | *Pterorhinus perspicillatus* (Gmelin, 1789) |
| Aves | Passeriformes | Muscicapidae | *Tarsiger cyanurus* (Pallas, 1773) |
| Aves | Passeriformes | Paridae | *Machlolophus spilonotus* (Bonaparte, 1850) |
| Aves | Passeriformes | Pellorneidae | *Schoeniparus brunneus* (Gould, 1863) |
| Aves | Passeriformes | Pittidae | *Hydrornis soror* (Wardla, 1881) |
| Aves | Passeriformes | Timaliidae | *Pomatorhinus ruficollis* (Hodgson, 1836) |
| Aves | Passeriformes | Turdidae | *Geokichla citrina* (Latham, 1790) |
| Aves | Passeriformes | Zosteropidae | *Zosterops simplex* (Swinhoe, 1861) |
| Mammalia | Piciformes | Megalaimidae | *Psilopogon faber* (Swinhoe, 1870) |
| Mammalia | Piciformes | Picidae | *Chrysophlegma flavinucha* (Gould, 1834) |
| Mammalia | Primates | Cercopithecidae | *Macaca mulatta* (Zimmermann, 1780) |
| Mammalia | Rodentia | Muridae | *Rattus norvegicus* (Berkenhout, 1769) |
| Mammalia | Rodentia | Sciuridae | *Callosciurus erythraeus* (Pallas, 1779) |
| Mammalia | Rodentia | Sciuridae | *Dremomys pyrrhomerus* (Thomas, 1895) |
| Mammalia | Rodentia | Sciuridae | *Ratufa bicolor* (Sparrman, 1778) |
| Mammalia | Rodentia | Sciuridae | *Tamiops maritimus* (Bonhote, 1900) |
| Mammalia | Scandentia | Tupaiidae | *Tupaia belangeri* (Wagner, 1841) |
| Mammalia | Strigiformes | Strigidae | *Glaucidium cuculoides* (Vigors, 1831) |
| Mammalia | Trogoniformes | Trogonidae | *Harpactes erythrocephalus* (Gould, 1834) |


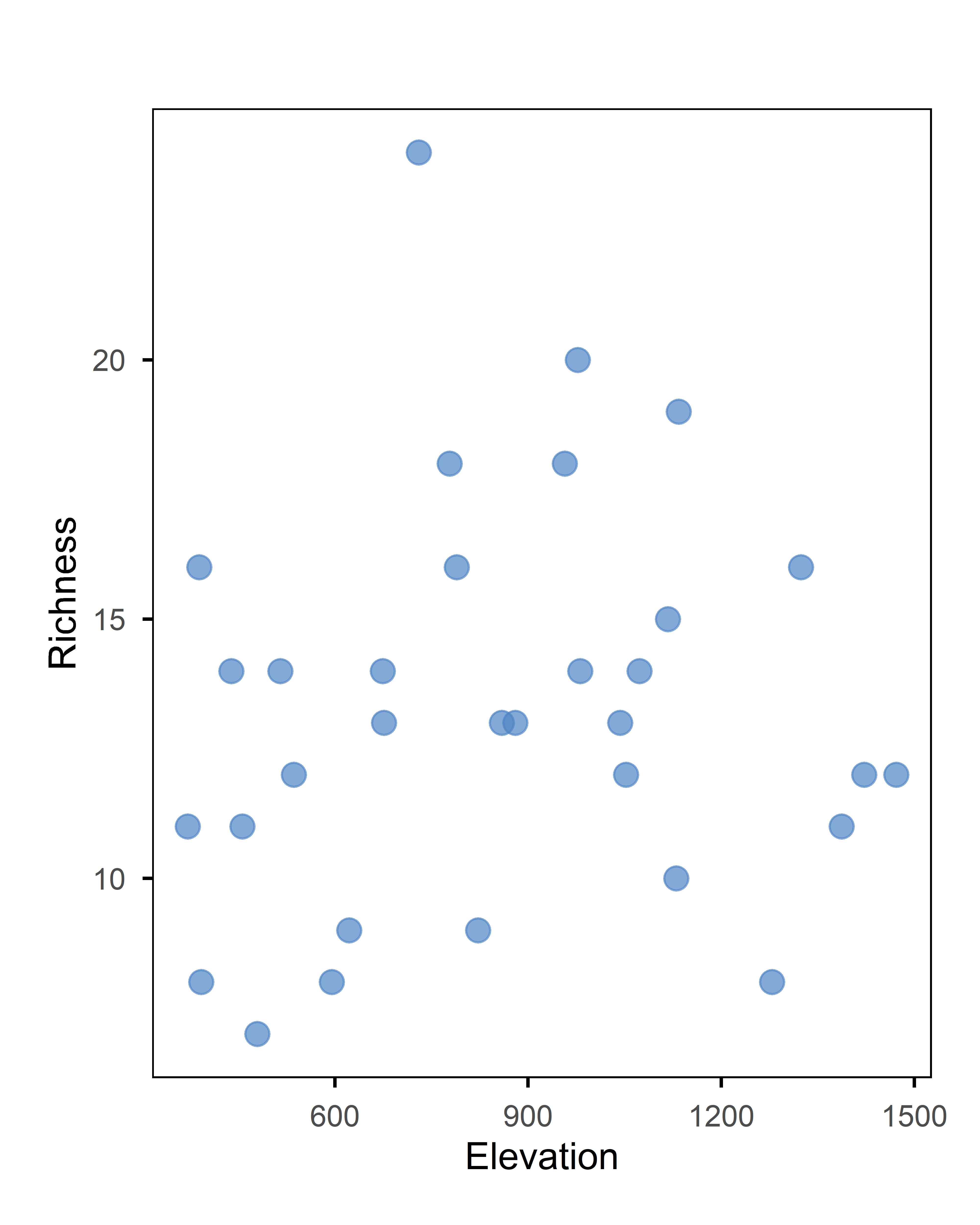


FIGURE S2 Scarabaeoidea species richness of 32 sampling plots along the elevational gradient in the Bawangling mountain.


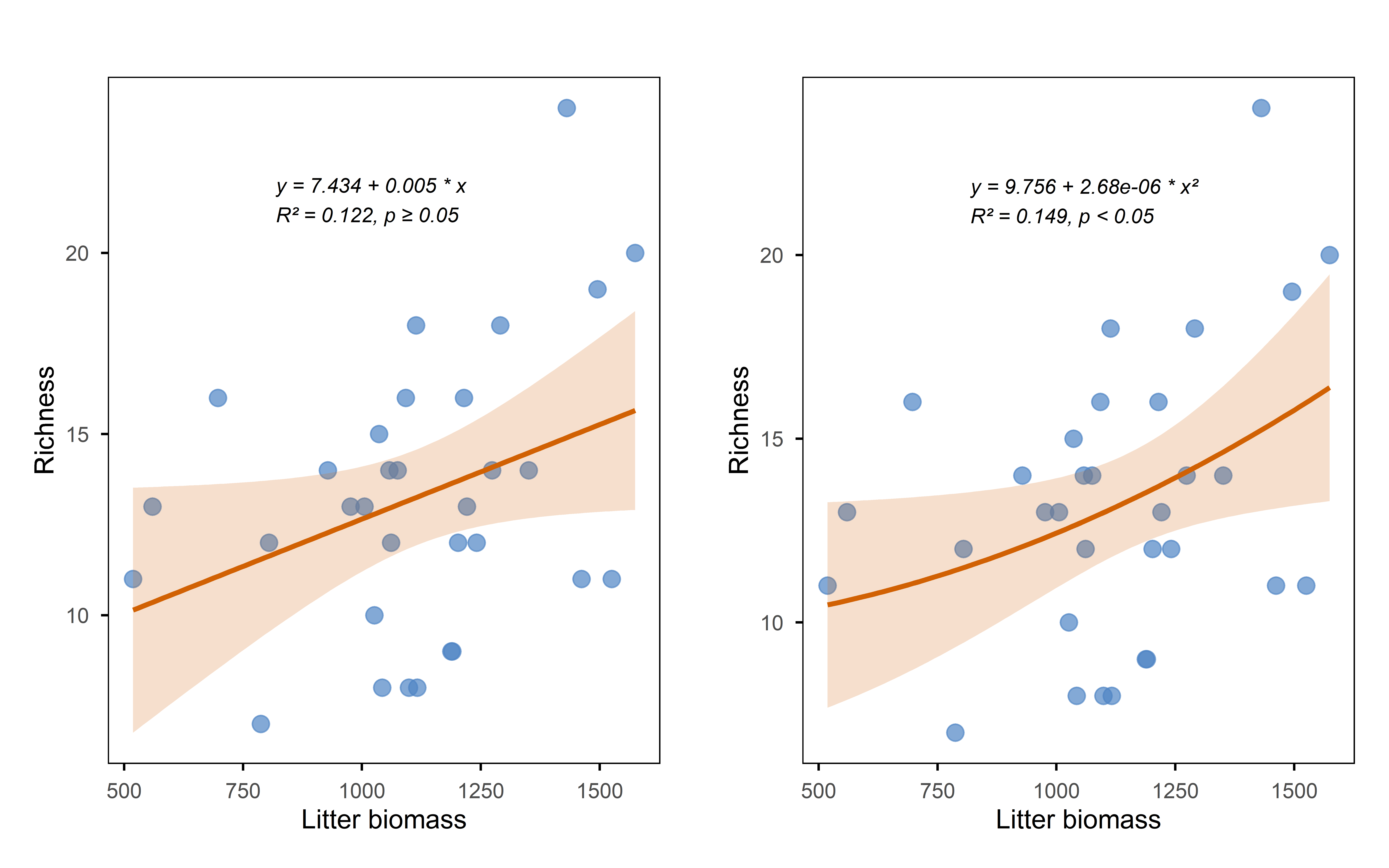


FIGURE S3 Relationships between Scarabaeoidea species richness and litter biomass. The shaded area indicates the 95% confidence interval.

Table S4 The comparison of results from simple linear models for Scarabaeoidea species richness and each potential driver along the elevational gradient in the Bawangling mountain with and without applying abundance threshold of three individuals.

| Model | Without abundance threshold of three individuals | | | |
| --- | --- | --- | --- | --- |
|  | Coefficient | p | R² | AICc |
| Species richness~Elevation | 0.002 | 0.310 | 0.036 | 181.6 |
| Species richness~Temperature | -0.495 | 0.321 | 0.034 | 179.1 |
| Species richness~Humidity | 11.180 | 0.479 | 0.017 | 182.2 |
| Species richness~Litter | 0.005 | **0.048** | 0.112 | 179.1 |
| Species richness~Predator richness | 0.001 | 0.839 | 0.419 | 221.0 |
| Species richness~Predator abundance | -0.012 | 0.331 | 0.033 | 181.7 |
| Model | With abundance threshold of three individuals | | | |
|  | Coefficient | p | R² | AICc |
| Species richness~Elevation | 0.002 | 0.435 | 0.021 | 177.6 |
| Species richness~Temperature | -0.341 | 0.463 | 0.018 | 177.6 |
| Species richness~Humidity | 11.312 | 0.441 | 0.021 | 177.6 |
| Species richness~Litter | 0.005 | **0.005** | 0.122 | 174.1 |
| Species richness~Predator richness | -0.132 | 0.381 | 0.026 | 177.4 |
| Species richness~Predator abundance | -0.006 | 0.587 | 0.010 | 177.9 |

Table S5 Model selection results from linear models of relationships between Scarabaeoidea species richness and potential drivers along the elevational gradient in the Bawangling mountain based on corrected Akaike Information Criterion (AICc) without applying abundance threshold of three individuals.

| Model | AICc | ΔAICc | Weight |
| --- | --- | --- | --- |
| Litter | 179.1 | 0.00 | 0.252 |
| Null model | 180.3 | 1.25 | 0.135 |
| Litter + temperature | 180.9 | 1.86 | 0.100 |

Table S6 Results of generalized linear models with Poisson and negative binomial distributions for relationships between Scarabaeoidea abundance and each potential predictor with abundance threshold of three individuals.

| Predictor | Poisson | | | |
| --- | --- | --- | --- | --- |
|  | Coefficient | p | R² | AICc |
| Elevation | -0.001 | <0.001 | 0.054 | 23696.070 |
| Temperature | 0.147 | <0.001 | 0.052 | 23750.384 |
| Humidity | -2.796 | <0.001 | 0.019 | 24559.382 |
| Predator richness | 0.012 | <0.001 | 0.003 | 24946.372 |
| Predator abundance | 0.001 | <0.001 | 0.008 | 24830.394 |
| Litter | 0.001 | <0.001 | 0.058 | 23579.724 |
| Predictor | Negative binomial | | | |
|  | Coefficient | p | R² | AICc |
| Elevation | -0.002 | <0.001 | 0.123 | 482.135 |
| Temperature | 0.365 | <0.001 | 0.112 | 482.576 |
| Humidity | -4.376 | 0.223 | 0.026 | 485.828 |
| Predator richness | 0.012 | 0.755 | 0.003 | 486.658 |
| Predator abundance | 0.002 | 0.572 | 0.008 | 486.477 |
| Litter | 0.001 | 0.078 | 0.064 | 484.420 |

Table S7 Results of generalized linear models with Poisson and negative binomial distributions for relationships between Scarabaeoidea abundance and each potential predictor without abundance threshold of three individuals.

| Predictor | Poisson | | | |
| --- | --- | --- | --- | --- |
|  | Coefficient | p | R² | AICc |
| Elevation | -0.001 | <0.001 | 0.054 | 23696.070 |
| Temperature | 0.147 | <0.001 | 0.052 | 23750.384 |
| Humidity | -2.796 | <0.001 | 0.019 | 24559.382 |
| Predator richness | 0.012 | <0.001 | 0.003 | 24946.372 |
| Predator abundance | 0.001 | <0.001 | 0.008 | 24830.394 |
| Litter | 0.001 | <0.001 | 0.058 | 23579.724 |
| Predictor | Negative binomial | | | |
|  | Coefficient | p | R² | AICc |
| Elevation | -0.002 | <0.001 | 0.123 | 482.135 |
| Temperature | 0.365 | 0.001 | 0.112 | 482.576 |
| Humidity | -4.376 | 0.223 | 0.026 | 485.828 |
| Predator richness | 0.012 | 0.755 | 0.003 | 486.658 |
| Predator abundance | 0.002 | 0.572 | 0.008 | 486.477 |
| Litter | 0.001 | 0.078 | 0.064 | 484.420 |


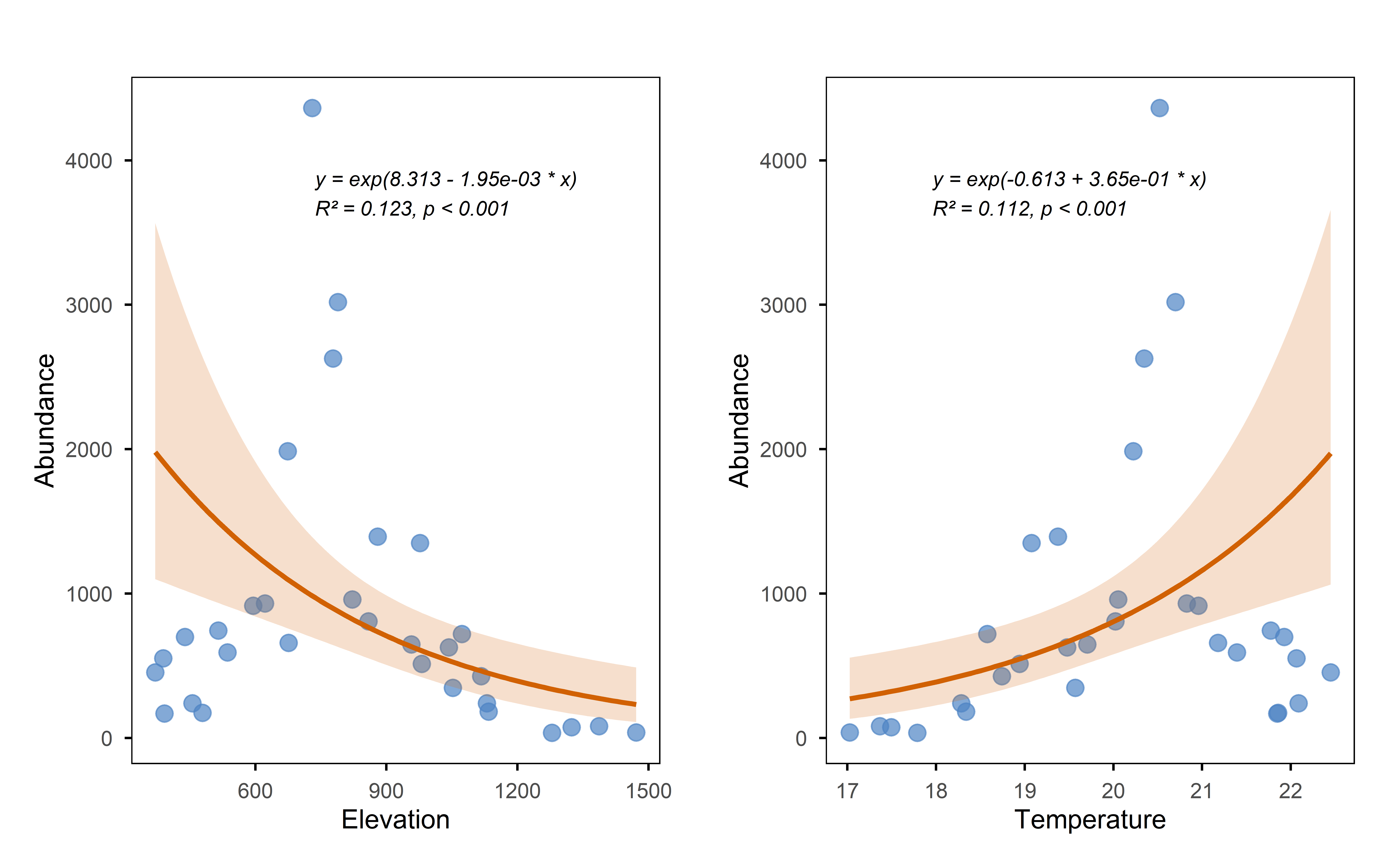
FIGURE S4 Relationships between Scarabaeoidea abundance and potential drivers (using negative binomial distribution). The shaded area indicates the 95% confidence interval.

Table S8 The comparison of results from generalized additive models (GAMs) of relationships between each potential driver and Scarabaeoidea abundance along the elevational gradient in the Bawangling mountain based on corrected Akaike Information Criterion (AICc) with and without applying abundance threshold of three individuals. The estimated degree of freedom (edf) of each model was provided.

| Model | Without abundance threshold of three individuals | | | |
| --- | --- | --- | --- | --- |
|  | edf | p | R² | AICc |
| Abundance~Elevation | 4.905 | <0.001 | 0.551 | 511.1 |
| Abundance~Temperature | 4.715 | 0.002 | 0.478 | 511.1 |
| Abundance~Humidity | 1.680 | 0.475 | 0.023 | 519.3 |
| Abundance~Litter | 1.000 | 0.279 | 0.007 | 518.3 |
| Abundance~Predator richness | 1.000 | 0.634 | -0.025 | 519.6 |
| Abundance~Predator abundance | 1.000 | 0.571 | -0.022 | 519.5 |
| Model | With abundance threshold of three individuals | | | |
|  | edf | p | R² | AICc |
| Abundance~Elevation | 4.916 | <0.001 | 0.552 | 511.1 |
| Abundance~Temperature | 4.747 | 0.003 | 0.480 | 511.1 |
| Abundance~Humidity | 1.857 | 0.476 | 0.023 | 518.3 |
| Abundance~Litter | 1.000 | 0.279 | 0.007 | 518.3 |
| Abundance~Predator richness | 1.000 | 0.632 | -0.025 | 519.6 |
| Abundance~Predator abundance | 1.000 | 0.569 | -0.022 | 519.5 |

Table S9 Model selection results from generalized additive models (GAMs) of relationships between potential drivers and Scarabaeoidea abundance based on corrected Akaike Information Criterion (AICc) without applying abundance threshold of three individuals.

| Model | AICc | ΔAICc | Weight |
| --- | --- | --- | --- |
| Temperature | 511.1 | 0.00 | 0.184 |
| Temperature + litter | 512.2 | 1.14 | 0.104 |
| Temperature + humidity | 512.7 | 1.65 | 0.081 |





FIGURE S5 Mantel tests between Scarabaeoidea β and elevation, temperature and predator β. The shaded area indicates the 95% confidence interval.

Table S10 Mantel correlations between assemblage dissimilarity matrices (β_sim_, β_sne_, and β_sor_) and distance matrices of potential drivers without applying abundance threshold of three individuals. *, p < 0.05; **, p < 0.01; ***, p < 0.001.

|  | β_sim_ | β_sne_ | β_sor_ |
| --- | --- | --- | --- |
| Elevation | 0.456^***^ | -0.163^***^ | 0.507^***^ |
| Temperature | 0.429^***^ | -0.159^***^ | 0.469^***^ |
| Humidity | 0.085 | 0.008 | 0.125^*^ |
| Litter | -0.102 | 0.041 | -0.094 |
| β of predators | 0.112 | 0.009 | 0.273^**^ |
| Predator abundance | -0.030 | -0.054 | -0.106 |
